# Overall and substance use-specific healthcare utilization among individuals with and without criminal justice involvement in Ontario, Canada

**DOI:** 10.1108/IJOPH-06-2024-0034

**Published:** 2025-06-13

**Authors:** Cayley Russell, Alexa Yakubovich, Patricia O’Campo, Kathleen Qu, Lesley Plumptre, Fiona Kouyoumdjian, Flora I. Matheson

**Affiliations:** Centre for Addiction and Mental Health, Institute for Mental Health Policy Research, Toronto, Canada, and Ontario Node, Canadian Research Initiative in Substance Matters (CRISM), Toronto, Canada; Department of Community Health and Epidemiology, Dalhousie University, Truro, Canada, and MAP Center for Urban Health Solutions, St Michael’s Hospital, Toronto, Canada; Center for Urban Health Solutions, St Michael’s Hospital, Toronto, Canada; Institute for Clinical Evaluative Sciences, Toronto, Canada; Department of Family Medicine, McMaster University, Hamilton, Canada; MAP Centre for Urban Health Solutions, St Michael’s Hospital, Toronto, Canada, and Dalla Lana School of Public Health, University of Toronto, Toronto, Canada

**Keywords:** Addiction, Canada, Correctional populations, Criminal justice system involvement, Health-care utilization, Overdose, Public health, Substance use

## Abstract

**Purpose:**

Correctional populations have higher rates of substance use disorders and related healthcare visits relative to the general population. However, limited evidence on substance use-related healthcare visits exists among this population. Using population data for Ontario, Canada, this study aims to examine overall and substance use-specific healthcare visits for individuals with and without known provincial criminal justice system involvement (CJI versus non-CJI, respectively).

**Design/methodology/approach:**

This retrospective study compared overall and substance use-related healthcare visits between April 1, 2015 and March 31, 2020 among provincially-incarcerated individuals (CJI group) versus those without criminal justice involvement (non-CJI group). Both groups were identified through available health administrative data and were individually matched by age, sex and material deprivation.

**Findings:**

The authors identified and matched 208,188 individuals (59.9% male) with and without CJI and a healthcare visit. Compared to the non-CJI group, those with CJI had approximately 20 times the rate of healthcare visits for alcohol use, drug use and illicit drug-related overdoses. Among those with CJI, females had a higher prevalence of overall healthcare visits, whereas males had a higher prevalence of substance use-specific visits.

**Research limitations/implications:**

Findings highlight the high number of healthcare visits for substance use-related needs among individuals with CJI in Ontario. These results can inform efforts to enhance correctional release planning, improve access to community-based treatment and strengthen substance use prevention and treatment interventions for this high-risk population.

**Practical implications:**

Results can inform efforts to enhance correctional release planning, improve access to community-based treatment, and strengthen substance use prevention and treatment interventions for this high-risk population.

**Originality/value:**

To the best of the authors’ knowledge, this study is the first in Canada to draw on population-level administrative health data to identify and match a large sample of individuals with and without CJI and examine substance use-specific healthcare utilization, longitudinally.

## Background

Criminal justice populations experience elevated rates of substance use disorder (SUD) diagnoses and related risks, however, in Canada, there are no reliable national SUD prevalence estimates (Bodkin *et al.*, 2021; Garrel and Farrell MacDonald, 2019; Mullins *et al.*, 2013). Extant data suggest that between 2010 and 2014, nearly four out of five individuals admitted to federal prisons in Canada reported a substance use issue, with high rates of polysubstance and injection drug use (Kelly and Farrell MacDonald, 2015a, 2015b). In the three-year period leading up to incarceration between 2002 and 2011, among individuals experiencing their first federal prison sentence, drug use was approximately 15 times more prevalent among incarcerated women compared to a matched comparison group of non-incarcerated women (47% vs 3%, respectively); prevalence was also significantly higher among women compared to their incarcerated male counterparts (47% vs 33%) (Butsang *et al.*, 2023). An Ontario-based study similarly found that individuals released from provincial jail in 2010 had an increased prevalence of SUDs compared to a general population matched control group (16.9% vs 1.3%, respectively, or a relative rate of 5.23) (Kouyoumdjian *et al.*, 2018a). Recent provincial data from British Columbia further suggest that the proportion of people admitted to jail with co-occurring mental health and SUDs has increased in the past 15 years (from 15% to 32%; 2009–2017) (Butler *et al.*, 2022).

As is the case with SUD prevalence data, healthcare utilization data among correctional populations in Canada are also scarce, despite the need for these data to inform healthcare services both in custody as well as during the community transition period (Kouyoumdjian *et al.*, 2016). Available Canadian provincial data suggest that individuals released from Ontario provincial jails in 2010 had significantly higher rates of both in-prison and post-release healthcare utilization compared to the general population (Kouyoumdjian *et al.*, 2018a). Specifically, individuals released from jail had nearly four times the relative rate of primary care utilization compared to a matched control group during the period immediately post-release, which remained elevated at two years post-release (Kouyoumdjian *et al.*, 2018b). The post-release period is a time of high risks for adverse health outcomes such as injuries, relapse, overdose and mortality (including premature mortality) (Binswanger *et al.*, 2013; Binswanger *et al.*, 2012; Butler *et al.*, 2023a; Groot *et al.*, 2016; Skinner and Farrington, 2020). Healthcare utilization in this period is complicated; Ontario-based data indicate that less than 40% of individuals released from provincial jail visited a family physician within two years post-release, and only 15% accessed Ontario’s Family Health Teams (i.e. interdisciplinary health professionals including social workers, dieticians, pharmacists, etc.) (Kouyoumdjian *et al.*, 2019). However, these individuals also experienced elevated emergency department (ED) usage, especially for acute complications. Furthermore, ED rates were particularly elevated among women compared to men, especially within the first week post-release, with women representing a crude rate of 3.3 visits per person-years compared to 2.5 visits per person-years among men (Tuinema *et al.*, 2020). Sex differences were also evident in primary care utilization among individuals released from provincial jail in Ontario in 2010, such that more women sought health care compared to men within the first three months post-release (Kouyoumdjian *et al.*, 2018b).

Given the high prevalence of SUDs and rates of healthcare utilization among correctional populations in Ontario, it follows that they likely experience elevated substance use-related hospitalizations. It further follows that there are likely key sex and/or gender differences in substance use-specific healthcare utilization, particularly compared to the general public. However, there are no available data on any of these indicators in the Canadian context, and in the province of Ontario, correctional health data are not readily available or accessible (Kouyoumdjian and Orkin, 2020). This is partly due to the separation between federal and provincial/territorial correctional jurisdictions in Canada. Specifically, the Correctional Service of Canada is responsible for the health care (and costs thereof) of people serving sentences of two or more years in federal institutions (otherwise known as prisons). On the other hand, The Ontario Ministry of Solicitor General is responsible for the supervision of people within provincial institutions (otherwise known as jails or detention/correctional centers) who are on remand (e.g. pretrial detention) or sentenced to less than two years, and health care is provided to these individuals as part of Ontario Health Insurance Plan (OHIP) (Murphy *et al.*, 2024). As such, there is no standardized data source that contains both federal and provincial correctional health data as they are collected and held separately. Furthermore, Ontario provincial correctional health data are historically paper-based (John Howard Society of Ontario, 2016b; Murphy and Sapers, 2020) and are independently collected by each of the 25 provincial institutions across the province, making it difficult to extract and link these data systematically; these data are in the process of moving to an electronic medical record database, but this has not yet been finalized (Ontario Ministry of the Solicitor General, 2024; Research Department, Ontario Ministry of the Solicitor General, 2023).

Therefore, there is limited information on healthcare utilization among correctional populations in Ontario. To address this gap, the present study used Ontario provincial administrative population health data linkages to examine overall and substance use-specific healthcare utilization among individuals who received healthcare services while in provincial custody (i.e. jail) or were referred from provincial jails to hospital-based services. These individuals were then matched to a general population sample without criminal justice system involvement who had healthcare visits within the same timeframe.

This article presents findings on the prevalence and relative rate of overall and substance use-specific healthcare visits by criminal justice involvement (CJI) status and sex. These findings can inform correctional release planning, enhance community-based treatment programs, and support best practices for ensuring continuity of care between correctional institutions and community healthcare settings in Ontario.

## Methods

### Study design and setting

We conducted a retrospective study of adults in Ontario, Canada, with and without known provincial criminal justice involvement (CJI vs non-CJI), who had a healthcare visit between April 1 2015 and March 31 2020, using secondary administrative data. CJI was defined those who were incarcerated in a provincial jail during the exposure period.

### Data sources and linkage

This study drew on data obtained from five databases housed within ICES, which is an independent, nonprofit research institute whose legal status under Ontario’s health information privacy law allows it to collect and analyze health care and demographic data, without consent, for health system evaluation and improvement. Databases included:

- the Canadian Institute for Health Information (CIHI) Discharge Abstract Database (DAD; for hospital admissions);
- the Registered Persons Database (RPDB; a registry that houses basic demographic information such as age, sex, location of residence, date of birth/death of all Ontario residents issued an OHIP number, used for health-care eligibility);
- the Ontario Mental Health Reporting System (OMHRS; for inpatient mental health admissions);
- the CIHI National Ambulatory Care Reporting System (NACRS; for ED visits); and
- OHIP, which contains information on physician visits (Government of Ontario, 2023) (see Appendix 1 for database details).

Participants were adult (18+) individuals in Ontario, Canada, with and without known CJI who had a healthcare visit within Ontario between April 1, 2015 and March 31, 2020. To determine CJI status, we identified healthcare visits that occurred between April 1, 2010 and March 31, 2015 (period of exposure to CJI) in which individuals were either referred from or accessed care in a provincial jail (CJI group) or not (non-CJI group). We then examined healthcare visits for both groups in the follow-up period (between April 1, 2015 and March 31, 2020).

Specifically, we determined CJI for people who had a valid ICES Identification Number (IKN) in the RPDB between April 1, 2010 and March 31, 2015, and who had a healthcare visit with an OHIP claim with the location specifying a provincial jail or a referral from one, based on the following criteria:

- where the referral was law enforcement (i.e. police, parole officer, court, jail) and the OHIP institution was a provincial jail in DAD (inpatient care);
- where the referral was a legal service (i.e. police, parole officer, court) and the OHIP institution was a provincial jail in NACRS (ED visits); and
- where the last digits of the referring facility was X65 in OMHRS (psychiatric inpatient care) and the OHIP institution was a provincial jail (outpatient care).

The non-CJI group was identified through ICES data for people with a valid ICES IKN in the RPDB between April 1, 2010 and March 31, 2015, and who had a healthcare visit between April 1, 2015 and March 31, 2020 based on the following criteria:

- having an OHIP claim with medical specialty only (i.e. a specialization in medicine, excluding non-medical specializations such as dentistry, physiotherapy, chiropractic, optometry, etc.);
- inpatient and same day surgery visits in DAD;
- ED visits in NACRS; and
- mental health admissions in OMHRS.

Individuals were excluded from the group if, based on RPDB records, as of April 1, 2020, they were a non-Ontario resident and had an invalid age (<18 or >105), had invalid or missing data for sex and date of birth, were deceased on or before the end of March 31, 2020 and the date of last contact in the healthcare system was not within the past five years from April 1, 2010.

The group underwent 1:1 matching for the CJI to the non-CJI group by exact age and neighborhood material deprivation quintile, separately for each of males and females. Neighborhood material deprivation reflects one of four dimensions of the Ontario Marginalization Index (van Ingen and Matheson, 2022). It is a measure of neighborhood material resources, which is associated with poor health and criminal justice involvement (Dennison and Swisher, 2019; Linton *et al.*, 2017; Matheson *et al.*, 2014). Residential postal codes were captured from the RPDB records to link individuals to their neighborhood of residence and assign area level material deprivation.

### Variable definitions

Our primary outcomes of interest were substance use-related healthcare visits which included visits specifically for drug use, alcohol use, overdose (any substance) and overdose (illicit drug-related). We also examined overall healthcare utilization patterns, including ambulatory care visits, ED visits, medical/surgical hospitalizations, mental health visits, visits for psychosis or depression, concurrent disorders (defined as any two or more of depression, psychosis and/or substance use), premature mortality (defined as mortality before age 60) and mortality, based on several sources including the RPDB. Outcomes were defined using International Classifications of Diseases (ICD) diagnosis codes where the diagnosis was indicated on any of the diagnostic codes on the record, or was defined using validated groups (see Appendix 2 Table A1 for variable codes) (Kim *et al.*, 2012; Quan *et al.*, 2008; Quan *et al.*, 2005).

### Statistical analysis

We performed descriptive analyses for proportions and frequencies of overall and substance use-specific healthcare visits among both the CJI and non-CJI groups, including *p*-values and standard deviations by CJI involvement and sex (i.e. males versus females within CJI group, and males versus females in non-CJI group), over the follow-up period (April 1, 2015 and March 31, 2020) (Tables 1–3). We also calculated incidence rate ratios (IRR) for both overall healthcare visits and substance use-specific visits, comparing the CJI to the non-CJI group (Table 4). IRRs were calculated using generalized estimating equations with negative binomial models. We used the logarithm of observed time in the study as an offset term to account for varying follow up times, accounting for censoring by death or OHIP eligibility. Person-time was calculated based on the total number of person years contributed by the individual while they were within Ontario and alive. Days of follow-up were defined from the date of the index record to either death date, end of OHIP eligibility, or March 31, 2020, whichever came first. Over 90% of each group was observed for the entirety of the five-year-follow-up. As expected, slightly more controls (non-CJI) were observed for the full period compared to cases (CJI). Of the 9% who did not have a full five-year-follow-up, >95% were observed for at least 2.5 years; <5% were observed for less than half the follow up period or 0.004% of the total group.

**Table 1:** Sample characteristics by sex and CJI status, April 1, 2015 to March 31, 2020

Criminal justice involvement (CJI) *N* (%)					No criminal justice involvement (Non-CJI) *N* (%)			
Age group	Total *n* = 208,188%	Females *n* = 83,295 (40.0%)	Males *n* = 124,893 (59.9%)	*p*-value	Total *n* = 208,188	Females *n* = 83,295 (40.0%)	Males *n* = 124,893 (59.9%)	*p*-value
18–30	61,042 (29.3)	21,544 (25.9)	39,498 (31.6)	<0.001	61,042 (29.3)	21,544 (25.9)	39,498 (31.6)	<0.001
31–40	41,857 (20.1)	15,144 (18.2)	26,713 (21.4)	<0.001	41,857 (20.1)	15,144 (18.2)	26,713 (21.4)	<0.001
41–50	39,324 (18.9)	15,450 (18.5)	23,874 (19.1)	<0.001	39,324 (18.9)	15,450 (18.5)	23,874 (19.1)	<0.001
51–60	34,268 (16.5)	14,689 (17.6)	19,579 (15.7)	<0.001	34,268 (16.5)	14,689 (17.6)	19,579 (15.7)	<0.001
61–70	17,981 (8.6)	8,771 (10.5)	9,210 (7.4)	<0.001	17,981 (8.6)	8,771 (10.5)	9,210 (7.4)	<0.001
71+	13,716 (6.6)	7,697 (9.2)	6,019 (4.8)	<0.001	13,716 (6.6)	7,697 (9.2)	6,019 (4.8)	<0.001
*Rural* Yes No	15,633 (7.5) 192,548 (92.5)	4,864 (5.8) 78,428 (94.2)	10,769 (8.6) 114,120 (91.4)	<0.001	18,544 (8.9) 189,644 (91.1)	7,646 (9.2) 75,649 (90.8)	10,898 (8.7) 113,995 (91.3)	<0.001

**Note(s):** *p-values are comparing within group sex differences

**Source(s):** Table by authors

**Table 2:** Overall healthcare visits by sex and CJI status, April 1, 2015 to March 31, 2020

Criminal justice involvement (CJI) *N* (%)					No criminal justice involvement (Non-CJI) *N* (%)			
Type of visit	Total *n* = 208,188	Females *n* = 83,295 (40)	Males *n* = 124,893 (59.9)	*p*-value	Total *n* = 208,188	Females *n* = 83,295 (40)	Males *n* = 124,893 (59.9)	*p*-value
Ambulatory care	195,924 (94.1)	81,128 (97.4)	114,796 (91.9)	<0.001	193,243 (92.8)	79,203 (95.1)	114,040 (91.3)	<0.001
ED visits	157,712 (75.8)	63,368 (76.1)	94,344 (75.5)	<0.001	121,396 (58.3)	50,689 (60.9)	70,707 (56.6)	<0.001
Medical/surgical hospitalizations	90,367 (43.4)	43,219 (51.9)	47,148 (37.8)	<0.001	72,557 (34.9)	36,020 (43.2)	36,537 (29.3)	0.028
Mental health hospitalizations	27,635 (13.3)	10,197 (12.2)	17,438 (14.0)	<0.001	3,327 (1.6)	1,333 (1.6)	1,994 (1.6)	0.946
Psychosis	16,835 (8.1)	5,684 (6.8)	11,151 (8.9)	0.003	1,248 (0.6)	416 (0.5)	832 (0.7)	<0.001
Depression	19,656 (9.4)	8,504 (10.2)	11,152 (8.9)	<0.001	3,897 (1.9)	1,771 (2.1)	2,126 (1.7)	<0.001
Concurrent disorders	14,227 (6.8)	4,803 (5.8)	9,424 (7.5)	0.006	1,041 (0.5)	338 (0.4)	703 (0.6)	<0.001
Mortality	13,058 (6.3)	4,504 (5.4)	8,554 (6.8)	<0.001	6,315 (3.0)	2,802 (3.4)	3,513 (2.8)	<0.001
Premature mortality	5,987 (2.9)	1,596 (1.9)	4,391 (3.5)	<0.001	1,292 (0.6)	410 (0.5)	882 (0.7)	<0.001

**Note(s):** *p-values are comparing within group sex differences

**Source(s):** Table by authors

**Table 3:** Substance use-specific healthcare visits by sex and CJI status, April 1, 2015 – March 31, 2020

	Criminal justice involvement (CJI) *N* (%)				No criminal justice involvement (Non-CJI) *N* (%)			
Type of visit	Total *n* = 208,188	Females *n* = 83,295 (40)	Males *n* = 124,893 (59.9)	*p*-value	Total *n* = 208,188	Females *n* = 83,295 (40)	Males *n* = 124,893 (59.9)	*p*-value
Alcohol use	22,080 (10.6)	6,397 (7.7)	15,683 (12.6)	<0.001	3,257 (1.6)	832 (1.0)	2,425 (1.9)	<0.001
Drug use	25,443 (12.2)	7,309 (8.8)	18,134 (14.5)	<0.001	2,422 (1.2)	696 (0.8)	1,726 (1.4)	<0.001
Overdose (any substance)	44,986 (21.6)	14,577 (17.5)	30,409 (24.3)	<0.001	8,978 (4.3)	3,505 (4.2)	5,473 (4.4)	0.055
Overdose (illicit drug-related)	14,227 (6.8)	4,803 (5.8)	9,424 (7.5)	<0.001	2,093 (1.0)	768 (0.9)	1,325 (1.1)	<0.001

**Note(s):** *p-values are comparing within group sex differences

**Source(s):** Table by authors

**Table 4:** Incidence rate ratios (IRR) for overall and substance use-specific healthcare visits sex and CJI status, April 1, 2015 to March 31, 2020

Type of visit	Overall IRR (95% CI)	Females IRR (95% CI)	Males IRR (95% CI)
*Overall healthcare visits*			
Ambulatory care visits	1.7 (1.7–1.7)	1.6 (1.5–1.6)	1.9 (1.9 −1.9)
ED visits	2.6 (2.5–2.6)	2.2 (2.1–2.2)	2.9 (2.8–2.9)
Medical/surgical hospitalizations	1.6 (1.6–1.6)	1.5 (1.5–1.5)	1.7 (1.7–1.8)
Mental health hospitalizations	14.4 (13.9–14.9)	14.1 (13.3–14.9)	14.5 (13.9–15.2)
*Substance use-specific healthcare visits*			
Alcohol use	19.1 (18.3–19.9)	23.3 (21.6–25.3)	17.9 (17.1–18.8)
Drug use	21.9 (21.0–22.8)	24.5 (22.6–26.5)	20.9 (19.9–21.9)
Overdose (any substance)	14.1 (13.7–14.5)	11.4 (10.8–11.9)	15.6 (15.1–16.1)
Overdose (illicit drug-related)	19.5 (18.7–20.4)	17.2 (15.9–18.5)	20.7 (19.6–21.8)

**Note(s):** *Non-CJI is reference group. All *p* values <0.0001

**Source(s):** Table by authors

All statistical analyses had an *a priori* statistical significance level of *p* ≤ 0.05, and were performed at ICES using SAS Enterprise Guide software, version 7.1 (SAS Institute, 2018). The full data set creation plan is available from the authors upon request. Data sets were linked using unique encoded identifiers and analyzed at ICES. This study was approved by the Research Ethics Board at Unity Health Toronto, Toronto, Canada [REB# 16–200].

## Results

### Sample characteristics

Table 1 presents the sample’s characteristics by sex and CJI status. There was a total of 208,188 people with CJI and 208,188 matched individuals (non-CJI) with a healthcare visit in Ontario between April 1, 2015 and March 31, 2020, the majority of whom were males (59.9%). Most individuals were between the ages of 18 and 30, and resided in a non-rural location. Males were generally younger than females.

#### Differences in the prevalence of overall healthcare visits by criminal involvement.

Compared to the non-CJI group, a higher proportion of those with CJI used ambulatory care (94.1% vs 92.8%), had an ED visit (75.8% vs 58.3%) and had a medical/surgical hospitalization (43.4% vs 34.9%) over the five-year follow up period. Similarly, a higher proportion of those in the CJI group had a mental health-related hospitalization (13.3% vs 1.6%), including specifically for psychosis (8.1% vs 0.6%), depression (9.4% vs 1.9%) and concurrent disorders (6.8% vs 0.5%) compared to the non-CJI group. Furthermore, compared to the non-CJI group, a higher proportion of individuals with CJI experienced premature mortality (2.9% vs 0.6%) and mortality (6.3% vs 3.0%) (see Table 2).

Among those with CJI, compared to males, more females had an ambulatory care visit (97.4% vs 91.9%; *p* ≤  0.001), an ED visit (76.1% vs 75.5%; %; *p* ≤  0.001), a medical/surgical hospitalization (51.9% vs 37.8%; *p* ≤  0.001) and a health-care visit for depression (10.2% vs 8.9%; *p* ≤  0.001). Conversely, more males with CJI than females had a mental health hospitalization (14.0% vs 12.2%; *p* ≤  0.001), including for psychosis (8.9% vs 6.8%; *p* ≤  0.01) and concurrent disorders (7.5% vs 5.8%; *p* ≤  0.01), as well as premature mortality (3.5% vs 1.9%; *p* ≤  0.001) and mortality (6.8% 5.4%; *p* ≤  0.001). CJI females and males had consistently higher proportions of visits for mental health-related concerns and mortality than their matched sample comparators (descriptive only).

#### Differences in the prevalence of substance use-specific healthcare visits by criminal justice involvement.

In terms of substance use-specific healthcare visits, those with CJI had a substantially higher proportion of visits than the non-CJI group. Specifically, those with CJI had more visits for alcohol use (10.6% vs 1.6%), drug use (12.2% vs 1.2%), overdoses (any substance) (21.6% vs 4.3%) and illicit drug-related overdoses (6.8% vs 1.0%) (see Table 3).

When examining substance use-related healthcare visits by CJI status and sex, compared to their same-sex counterparts, both males and females with CJI had a higher proportion of all substance use-related healthcare visits the non-CJI group. Specifically, compared to non-CJI males, males with CJI had a higher proportion of substance use-related healthcare visits due to alcohol use (12.6% vs 1.9%), drug use (14.5% vs 1.4%), overdoses (any substance) (24.3% vs 4.4%) and illicit drug use overdoses (7.5% vs 1.1%). Similarly, compared to non-CJI females, females with CJI had a higher proportion of substance use-related healthcare visits for alcohol (7.7% vs 1.0%), drug use (8.8% vs 0.8%), overdoses (any substance) (17.5% vs 4.2%) and illicit drug overdoses (5.8% vs 0.9%) (see Table 3).

When examining substance use-related healthcare visits among those with CJI, males experienced more healthcare visits than females related to substance use, including for alcohol use (12.6% vs 7.7%; *p* ≤  0.001), drug use (14.5% vs 8.8%; *p* ≤  0.001), overdoses (any substance: 24.3% vs 17.5%; *p* ≤  0.001) and overdoses specifically related to illicit drugs (7.5% vs 5.8%; *p* ≤  0.001). These sex-specific trends were also reflected in the non-CJI group.

#### Differences in rates of overall and substance use-specific healthcare visits by criminal justice involvement.

Individuals with CJI had elevated rates of overall healthcare visits, as well as those specific to substance use, compared to the non-CJI group. Specifically, those with CJI had a higher rate of ambulatory care visits (1.7), ED visits (2.6), medical/surgical hospitalizations (1.6) and mental health-specific hospitalizations (14.4) compared to non-CJI individuals; rates were similar between sexes (see Table 4).

When examining substance use-related visits in particular, compared to those without CJI, the CJI group had substantially elevated rates of healthcare visits for alcohol use (19.1), drug use (21.9), overdose (any substance) (14.1) and illicit drug-related overdoses (19.5) (see Table 4). Furthermore, compared to non-CJI females, CJI females had elevated rates of healthcare visits for alcohol use (23.4) and drug use (24.5). Rates of overdoses (any substance and illicit drug-related) among CJI females were 11.4 and 17.2 times that of non-CJI females, respectively. Similarly, compared to non-CJI males, CJI males had increased rates of healthcare visits for alcohol use (17.9), drug use (20.9) and illicit drug-related overdoses (20.7). The rate of an overdose-related healthcare visit for any substance among CJI males (15.6) was also elevated compared to that of non-CJI males (see Table 4).

## Discussion

The current study examined overall and substance use-specific healthcare visits amongst those with CJI in Ontario, compared with a non-CJI matched sample. This is the first study in the Canadian context and one of few internationally to examine substance use-specific healthcare visits among those with CJI using population-level administrative health data and a matched comparison group. By using a sample that was matched on age, sex and material deprivation, this paper was able to demonstrate the significant impact of CJI on healthcare utilization for substance use-related issues, over and above these sociodemographic factors. Furthermore, our approach demonstrates an interesting and novel way to identify people with CJI exposure through health administrative data, given the lack of accessible health data among correctional populations in the province.

Our results reveal and reflect a myriad of challenges that those with CJI face regarding their health, particularly in comparison to individuals who have also accessed health care in Ontario but who were not identified as criminal justice-involved. For instance, compared to the matched general population sample, those with CJI had substantially higher overall healthcare visits, including ambulatory care utilization, ED visits, and medical/surgical hospitalizations. They also had more mental health conditions, such as depression, psychosis and concurrent disorders, and experienced a rate of mental health-related hospitalizations that was 14 times that of their non-CJI counterparts. Furthermore, individuals with CJI in Ontario had both a higher proportion and rate of all substance use-related hospitalizations compared to the matched general population sample. Specifically, those with CJI had rates of healthcare visits for alcohol use, drug use, and illicit drug-related overdoses that were approximately 20 times the rate of the general population. These data underscore the complexity of this population’s healthcare needs as well as the high utilization of the healthcare system overall.

Other studies have similarly demonstrated associations between mental health and addiction-specific healthcare utilization among correctional populations, aligning our study within the current literature. For instance, studies from the USA suggest that compared to those without CJI, those with CJI have higher rates of medical, mental health and substance use-related ED visits and hospitalizations (Shah *et al.*, 2024; Jue *et al.*, 2024), as well as a higher frequency of ED visits, including visits that result in hospitalizations (McConville *et al.*, 2018). In Ontario, studies have confirmed high levels of mental health and addiction healthcare use prior to, during, and post-release among those with CJI (Kurdyak *et al.*, 2022; Lebenbaum *et al.*, 2024; Vijh *et al.*, 2024). Furthermore, the link between incarceration and the risk for experiencing an overdose has been well established in the literature, including in the international (Binswanger, 2019; Brinkley-Rubinstein *et al.*, 2018; Gan *et al.*, 2021; Merrall *et al.*, 2010; Mital *et al.*, 2020; Victor *et al.*, 2022), national (Gan *et al.*, 2021) and Ontario-specific (Butler *et al.*, 2023a; Groot *et al.*, 2016; Matheson *et al.*, 2023) contexts.

Given the elevated rates of both overall and substance use-specific healthcare utilization among those with CJI, it is not surprising that correctional populations are among the highest-costing healthcare users. For instance, studies have found that individuals with CJI in Ontario have higher healthcare costs compared to those without CJI, with especially elevated costs among females compared to males, and among females with CJI compared to females without CJI (de Oliveira *et al.*, 2022). Sex differences were similarly apparent within our study, where both males and females had substantially elevated rates of substance use-related healthcare visits compared to their non-CJI same-sex counterparts. Specifically, females with CJI had rates of healthcare visits for alcohol and drug use that were nearly 25 times higher than females without CJI, whereas males with CJI had rates of healthcare visits for drug use and illicit drug-related overdoses that were 20 times that of males without CJI. Moreover, when examining overall healthcare utilization, both males and females with CJI had similarly elevated rates of ambulatory care and ED visits, as well as medical/surgical and mental health-related hospitalizations compared to their non-CJI same-sex counterparts.

Within-group sex differences were also apparent. Among those with CJI, females with CJI tended to have a higher proportion of general healthcare visits (ambulatory care, ED, medical/surgical, depression) compared to CJI males, whereas CJI males had higher rates of mental health and substance use-specific healthcare visits (mental health hospitalizations, psychosis, alcohol use, drug use and all overdose types) compared to CJI females. These findings are interesting and add to the complexity of understanding sex differences and the gendered nature of correctional system involvement and related outcomes. For instance, most of the available data suggest females with CJI have worse overall health statuses compared to males with CJI. Specifically, females with CJI are more likely than males to have elevated morbidity, psychiatric conditions, hospitalizations and ED visits, including for substance use, both prior to and post-release, and these visits are often considered high urgency (Butsang *et al.*, 2023; Norris *et al.*, 2021; Tuinema *et al.*, 2020). Our findings suggest there may be sex-specific differences in the specific types of healthcare needs, with females potentially requiring more acute/general care and males requiring more substance use-specific care. These results should be further teased apart in future research.

Overall, our findings corroborate extant literature describing the high healthcare-related needs and disproportionately greater rates of SUDs and healthcare utilization among individuals with CJI (Butler *et al.*, 2022; Kouyoumdjian *et al.*, 2018b; Kouyoumdjian *et al.*, 2018a), as well as the gendered nature of this risk (Butler *et al.*, 2023a; Fazel *et al.*, 2017; Krawczyk *et al.*, 2020). However, they also demonstrate that those with CJI are accessing healthcare for substance use-related issues, despite well-documented barriers to healthcare and treatment utilization among individuals with CJI (Hu *et al.*, 2020). For instance, a lack of knowledge or awareness of available supports, a lack of identification, difficulties navigating the healthcare system, the absence of a primary healthcare provider, and competing demands related to correctional release plans and/or basic needs (housing, employment, financial stability, social services) have all been documented as key challenges to healthcare utilization for individuals with CJI (Binswanger *et al.*, 2011; Kaplowitz *et al.*, 2023; Kouyoumdjian *et al.*, 2018b; Russell *et al.*, 2021b; Russell *et al.*, 2022; Salem *et al.*, 2021). Moreover, stigmatization and discrimination amongst healthcare workers and within healthcare settings (Fahmy *et al.*, 2018) has been identified as a main deterrent for seeking support (Frank *et al.*, 2014). Studies have also shown that healthcare discrimination based on CJI status increases the odds of poorer health outcomes (Redmond *et al.*, 2020).

Considering these known barriers, our data suggest that the overall and substance use-specific issues for which individuals with CJI are presenting to healthcare for are urgent, emphasizing the necessity for increased SUD prevention and treatment interventions for CJI populations. It is likely that many individuals present to the ED or are hospitalized due to the barriers described above, such as a lack of access to preventative care, including primary care physicians, low-barrier harm reduction and addiction treatment services, and support for basic needs (Kouyoumdjian *et al.*, 2018b; Kouyoumdjian *et al.*, 2019). It is also likely that these issues result in higher healthcare costs for this population (de Oliveira *et al.*, 2022).

As such, key recommendations can be drawn from our findings. Sex and gender-specific interventions should be explored, identifying the specific healthcare needs that each sex or gender may have, considering the key differences in types of healthcare presentations between sexes noted in our study. Suggestions that have been put forth include the need to increase information and education on the risks of SUDs, enhance sex/gender-specific tailored interventions, reduce stigmatization and improve discharge planning through linkages between correctional facilities and community-based healthcare providers (Ramaswamy and Freudenberg, 2022; Russell *et al.*, 2021b; Russell *et al.*, 2022). In particular, targeted interventions should be undertaken pre-community release to identify those most likely to be at an increased risk of health-related needs post-release (e.g. those with SUDs and high mental health comorbidity with impending release dates). For instance, there is a need to enhance correctional release planning by developing individualized discharge plans that include thorough assessments of substance use needs and risk factors. While the discharge planning process exists, research has demonstrated that many individuals in Ontario lack access to adequate discharge plans prior to release, which is associated with negative outcomes and compromised community reintegration (Hu *et al.*, 2020; John Howard Society of Ontario, 2016a; Russell *et al.*, 2021b). Therefore, the discharge planning process should be enhanced and designed in collaboration with a multi-disciplinary team of correctional staff, social workers, mental health professionals, peer support specialists, community health care providers and the individual’s family and support system. The discharge planning process should begin as early as possible and incorporate a hybrid approach, drawing on evidence-based and validated mental health and substance use screening tools as well as one-on-one interviews with individuals and their support systems, exploring their needs, goals, barriers and available supports (John Howard Society of Ontario, 2016a). Peer navigators can then be leveraged during the release process to support and guide individuals in navigating post-release transitions and connections to community health and social services, ensuring they are reaching the goals identified in their discharge plans (Enich *et al.*, 2023; Morse *et al.*, 2017; Tillson *et al.*, 2022).

In addition, community transition teams, which support discharge planning for those with mental health and addictions and provide case management should be drawn on to further support connections to community supports and services (Jordan *et al.*, 2022). These teams work together with individuals to serve as a key point of contact and to help monitor their progress through regular check-ins where they provide support, including with scheduling appointments with healthcare providers, substance use treatment programs, housing agencies and employment services, among other needs. In Ontario, there are a few programs of this nature, such as Community Reintegration Planning Tables, which is a partnership between the Ministry of the Solicitor General and the Ontario Provincial Human Services and Justice Coordinating Committee, intended to support a person-centered, multi-sectoral approach to reintegration planning for those with high needs being released from provincial jails in Ontario (Human Services and Justice Coordinating Committee (HSJCC), 2023). In addition, the Canadian Mental Health Association’s Release from Custody program similarly aims to support people with mental health and addictions to reintegrate into the community upon release from incarceration through short-term case management support (ConnexOntario, 2024). However, these programs are limited to specific jails and/or communities and their availability is not standardized across the province. In the US, the Transitions Clinic Network draws on a multidisciplinary team to provide connections to clinics that have been trained to serve the unique needs of this population (Transitions Clinic Network (TCN), 2022). This model has been shown to reduce re-incarceration, acute care visits (Shavit *et al.*, 2017), ED utilization (Wang *et al.*, 2012), length of hospital stays, as well as preventable hospitalizations (i.e. those for which primary care or early intervention could potentially prevent the need for hospitalization) among those involved in the program (Wang *et al.*, 2019). Furthermore, this program has demonstrated average monthly criminal justice system cost savings, with significantly lower costs for those involved compared to a matched group (Harvey *et al.*, 2022). Other cost-benefit analyses of corrections-community transition programs have also demonstrated increased cost savings (Spaulding *et al.*, 2013). Investments should therefore be directed into designing, implementing, and evaluating these types of programs in Ontario.

Furthermore, additional interventions that focus on continuity of care and seamless linkages to outpatient treatment services upon release for those with substance use needs in particular should be enhanced. Specifically, providing medications for opioid use disorder (MOUD) (e.g. methadone, buprenorphine/naloxone) pre-release has been proven to reduce release-related risks such as overdose and mortality, as well as healthcare utilization among individuals with SUD (Klemperer *et al.*, 2023; Lim *et al.*, 2023). For example, in the Canadian province of BC, individuals with CJI who were dispensed methadone had a 50% lower rate of hospitalization for any cause and a 30% lower rate of hospitalizations related specifically to a SUD (Russolillo *et al.*, 2019). As such, efforts to expand access to pre-release MOUD should be bolstered. Such efforts should minimally include the provision of MOUD prescriptions to individuals upon release to prevent treatment gaps during the community transition period. During the discharge planning process, individuals who require MOUD should be given take-home doses and provided with multi-day (or longer) prescriptions, which can reduce the burden of having to find and attend a MOUD clinic immediately upon release to obtain required medications (Russell *et al.*, 2021b). Long-acting injectable buprenorphine, which provides four-week or longer sustained buprenorphine serum levels has recently been approved in Canada, showing promise as a key bridge medication and should also be considered (Russell *et al.*, 2024; Scott *et al.*, 2021). Within the community, low barrier treatment options, such as walk-in addiction clinics, telehealth and mobile services that support individuals upon release should be expanded. Adopting the abovementioned strategies can help create a seamless, integrated approach to addressing SUDs and related harms among correctional populations in Ontario, ultimately improving long-term health outcomes and reducing the strain on the healthcare system.

### Study strengths and limitations

This study is a large, longitudinal, population-based study, identifying individuals with CJI using administrative health data (as opposed to self-report data) and examining their healthcare utilization over a five-year period, which is a major strength. In addition, the study uses a matched sample which ensures that the groups are similar in nature in terms of age, sex and material deprivation, and therefore provides greater certainty that our results demonstrate the impact of CJI as opposed to other potentially confounding variables. By matching the sample on age, sex, and material deprivation, we were able to better isolate the specific impact of CJI on substance use-related healthcare visits, which also allows for better generalizability and reliability of our findings. Furthermore, given that there is no standardized electronic medical record system that captures health data for all individuals involved in the criminal justice system in Ontario, this study leverages available administrative data linkages to explore the impact of CJI on substance use-related healthcare utilization, which has not been done in the Ontario context previously.

However, limitations of the current study must be noted. The CJI sample was derived from administrative health data based on codes related to referrals from provincial jails, police, courts and parole offices, rather than a sample derived from administrative criminal justice system records, as the latter are not easily accessible or available. This is a novel approach to derive the sample, but we were not able to verify the records against actual correctional data. It is also possible that people in the matched group may have experienced justice involvement at some time outside the study exposure period, which we could not account for; if individuals within the non-CJI sample did, indeed, have correctional system involvement at some point, they may therefore have been misclassified, but there is no way of knowing this for sure. In addition, it is important to note that by virtue of the creation of the sample, everyone had at least one healthcare visit in the reference period, which means the sample is potentially different from the general population in important ways for both the CJI and non-CJI groups. Specifically, our sample may be more likely to have healthrelated needs that brought them into contact with the healthcare system, which may have inflated our findings. Relatedly, the CJI group represents a unique population that is captive and highly monitored, reflecting inherent power imbalances and a loss of autonomy, particularly in regards to healthrelated decisions. As such, this status may have influenced individual-level health-\care utilization, however, it is unlikely this is the only factor at play.

Additional variables for matching the groups beyond age, sex, and deprivation were not available within the data set, nor were variables related to ethnicity/race as data on these variables are not comprehensive. In light of the over-representation of Black and Indigenous individuals within Canadian correctional institutions (Owusu-Bempah *et al.*, 2023; Sandulescu, 2021), this is a key limitation. Furthermore, sex was limited to a binary measure, and we were not able to capture or present information on gender, which is also a limitation given the key sex differences among those with CJI apparent within our study, as well as sex and gender differences associated with health among those with CJI more broadly in the literature (Butsang *et al.*, 2023). Moreover, the sex distribution of our sample is non-representative of the sex distribution of people who experience incarceration overall. Specifically, our CJI sample reflects a higher proportion of females who typically represent a minority (approximately 6% in Canada) of incarcerated individuals (Government of Canada, 2024). However, the ability to derive the sample from an administrative database enabled us to construct the sample with a larger proportion of females in the CJI group, enabling a more robust analysis. In general, based on the lack of available data on specific made-vulnerable sub-populations including racialized and gendered groups, we were not able to explore potential inequities in rates in healthcare visits among these groups. Going forward, research should focus on examining these inequities and include additional variables related to gender and race/ethnicity.

Although the use of a matched group strengthens our results and allows for greater generalizability, we can never be certain that other potential variables have not impacted the findings; it is unlikely we have accounted for all potential confounders. Also, in terms of external validity, our results are specific to the Ontario context, and as such, are unlikely to be generalizable to jurisdictions with substantially different healthcare and/or criminal justice systems. Furthermore, our CJI sample was limited to individuals who are incarcerated in provincial jails and excludes those in federal prison. However, in Ontario, the majority of people involved in the criminal justice system are incarcerated provincially (average daily in-custody counts are 22,318 provincial versus 12,667 federal) (Statistics Canada, 2024). Data were also collected prior to the COVID-19 pandemic and the corresponding increase and escalation of the overdose poisoning crisis in Ontario; rates of substance use-specific healthcare visits during and post-COVID-19 may have disproportionately impacted those with CJI (Butler *et al.*, 2023b; Imtiaz *et al.*, 2021; Russell *et al.*, 2021a). As such, substance use-related healthcare visits reported here may substantially underrepresent the current situation, where more people are experiencing SUD-related harms than ever before (Public Health Ontario, 2024). Moreover, healthcare visits do not necessarily equate to health problems, which are likely undercounted, and therefore an underestimation of the true effect of CJI on health-related needs. Future research should examine when substance use-related healthcare visits occur temporally in relation to their CJI, considering most of the available literature points to the post-release period as extremely high risk for health harms (Merrall *et al.*, 2010).

## Conclusion

In conclusion, our findings provide insights into the extent to which males and females who are incarcerated in provincial jails in Ontario access healthcare services in general, as well as specifically for substance use-related concerns. Results suggest an elevated rate of overall and substance-use specific healthcare visits amongst individuals with CJI in the province of Ontario, underscoring the specific impact of CJI on healthcare utilization. Those with CJI are 20 times more likely than those without CJI to require a healthcare visit related to alcohol, drug use, and illicit drug-related overdoses, and are 14 times more likely to experience a mental health-related visit. These findings emphasize the urgent need for more comprehensive and standardized systems and interventions to support the healthcare needs of this high risk population. More research is also required to illuminate and better understand sex differences in the risk of SUD and related health harms among those with CJI.

## Acknowledgement

This study was supported by ICES, which is funded by an annual grant from the Ontario Ministry of Health (MOH) and the Ministry of Long-Term Care (MLTC). This study was also supported by MAP Centre for Urban Health Solutions, St. Michael’s Hospital, Unity Health Toronto, and Dalla Lana School of Public Health, University of Toronto. This document used data adapted from the Statistics Canada Postal Code^OM^ Conversion File, which is based on data licensed from Canada Post Corporation and/or data adapted from the Ontario Ministry of Health Postal Code Conversion File, which contains data copied under license from ©Canada Post Corporation and Statistics Canada. Parts of this material are based on data and/or information compiled and provided by Canadian Institutes for Health Information (CIHI) and MOH. The analyses, conclusions, opinions and statements expressed herein are solely those of the authors and do not reflect those of the funding or data sources; no endorsement is intended or should be inferred. The authors thank the Toronto Community Health Profiles Partnership for providing access to the Ontario Marginalization Index. This study is also supported in part by funding from the Canadian Institutes for Health Research (CIHR; grant # REN-181677) for the Ontario Node of the Canadian Research Initiative in Substance Matters (CRISM); this funder had had no role in the design of this study, nor its execution, analyses, interpretation of data or publication.

*Funding*: This study was funded by MAP Centre for Urban Health Solutions, Unity Health Toronto No. N/A; ICES and the Ontario Ministry of Health and Long-Term Care No. N/A; Dalla Lana School of Public Health, University of Toronto No. N/A; Canadian Institutes of Health Research No. REN-181677.

*Conflicts of interest:* The authors have no conflicts of interest to declare.

*Data availability:* The data set from this study is held securely in coded form at ICES. While legal data sharing agreements between ICES and data providers (e.g. health-care organizations and government) prohibit ICES from making the data set publicly available, access may be granted to those who meet pre-specified criteria for confidential access, available at www.ices.on.ca/DAS (email: das@ices.on.ca). The full data-set creation plan and underlying analytic code are available from the authors upon request, understanding that the computer programs may rely upon coding templates or macros that are unique to ICES and are therefore either inaccessible or may require modification.

## Appendix 1. Databases

### Ontario Health Insurance Plan claims database

The OHIP claims database contains information on inpatient and outpatient services provided to Ontario residents eligible for the province’s publicly funded health insurance system by fee-for-service health-care practitioners (primarily physicians) and “shadow billings” for those paid through nonfee for service payment plans. The main data elements include patient and physician identifiers (encrypted), code for service provided, date of service, associated diagnosis and fee paid.

### Ontario Mental Health Reporting System

The OMHRS is compiled by the Canadian Institute for Health Information and contains administrative, clinical (diagnoses and procedures), demographic and administrative information for all admissions to adult designated inpatient mental health beds. This includes beds in general hospitals, provincial psychiatric facilities and specialty psychiatric facilities. Clinical assessment data is ascertained using the Resident Assessment Instrument for Mental Health (RAI-MH), but different amounts of information are collected using this instrument depending on the length of stay in the mental health bed. Multiple assessments may occur during the length of a mental health admission.

### Registered Persons Database files

The RPDB provides basic demographic information (age, sex, location of residence, date of birth and date of death for deceased individuals) for those issued an Ontario health insurance number. The RPDB also indicates the time periods for which an individual was eligible to receive publicly funded health insurance benefits and the best known postal code for each registrant on July 1 of each year.

### National Ambulatory Care Reporting System

The NACRS is compiled by the Canadian Institute for Health Information and contains administrative, clinical (diagnoses and procedures), demographic and administrative information for all patient visits made to hospital- and community-based ambulatory care centers (emergency departments, day surgery units, hemodialysis units and cancer care clinics). At ICES, NACRS records are linked with other data sources (DAD, OMHRS) to identify transitions to other care settings, such as inpatient acute care or psychiatric care.

### Discharge Abstract Database

The DAD is compiled by the Canadian Institute for Health Information and contains administrative, clinical (diagnoses and procedures/interventions), demographic and administrative information for all admissions to acute care hospitals, rehab, chronic and day surgery institutions in Ontario. At ICES, consecutive DAD records are linked together to form “episodes of care” among the hospitals to which patients have been transferred after their initial admission.

*Source(s):* By authors

## Appendix 2

Table A1

**Table A1:** Variable codes

*Appendix: Codes*	
Mental illness and substance use	Alcohol abuse ICD-9: 255.2, 291.1–291.3, 291.5–291.9, 303.0, 303.9, 305.0, 357.5, 425.5, 535.3, 571.0, 571.3, 980.x, v11.3 ICD-10: F10, E52, G62.1, I42.6, K29.2, K70.0, K70.3, K70.9, T51.X, Z50.2, Z71.4, Z72.1 OHIP: 291, 303, 304 OMHRS: any dsm axis 1 or axis2 code starting with 291 Drug Abuse ICD-9: 292.x, 304.x, 305.2–305.9, V65.42 ICD-10: F11.x-F16.x, F18.x, F19.x, Z71.5, Z72.2 OHIP: 304 OMHRS: any dsm axis 1 or axis 2 code starting with 292 Psychoses ICD-9: 293.8, 295.x, 296.04, 296.14, 296.44, 296.54, 297.x, 298.x ICD-10: F20.x, F22.x-F25.z, F28.x, F29.x, F30.2, F31.2, F31.5 OHIP: 293,295,297,298 OMHRS: dsm axis 1 or axis 2 codes 29381 or 29382, any code starting with 295,29710, and any code starting with 298 Depression ICD-9: 262.2, 296.3, 296.5, 300.4, 3009.x, 311 ICD-10: F20.4, F31.3-F31.5, F32.x, F33.x, F34.1, F41.2, F43.2 OHIP: 296, 311 OMHRS: dsm axis 1 or axis 2 codes 29620, 29621, 29622, 29623, 29624, 29625, 29626, 29630, 29631, 29632, 29633, 29634, 29635, 29636, 31100
Drug overdose	E850.0-E858.9; E930-E949; E980-E989; 960–979; [T36-T50; Y40-Y59; X60-X69] 292; 304; 305; [F10-F19; F55; Y45.0; Z50.3; Z71.5]

**Source(s):** Table by authors
